# Interplay between OXA-10 β-Lactamase Production and Low Outer-Membrane Permeability in Carbapenem Resistance in Enterobacterales

**DOI:** 10.3390/antibiotics12060999

**Published:** 2023-06-01

**Authors:** Isaac Alonso-García, Juan Carlos Vázquez-Ucha, Marta Martínez-Guitián, Cristina Lasarte-Monterrubio, Salud Rodríguez-Pallares, Pablo Camacho-Zamora, Soraya Rumbo-Feal, Pablo Aja-Macaya, Lucía González-Pinto, Michelle Outeda-García, Romina Maceiras, Paula Guijarro-Sánchez, María José Muíño-Andrade, Ana Fernández-González, Marina Oviaño, Concepción González-Bello, Jorge Arca-Suárez, Alejandro Beceiro, Germán Bou

**Affiliations:** 1 Servicio de Microbiología and Instituto de Investigación Biomédica A Coruña (INIBIC), Complexo Hospitalario Universitario A Coruña, As Xubias 84, 15006 A Coruña, Spain; isaac.alonso.garcia@sergas.es (I.A.-G.); juan.carlos.vazquez.ucha@sergas.es (J.C.V.-U.); cristina.lasarte.monterrubio@sergas.es (C.L.-M.); salud361@gmail.com (S.R.-P.); pablo.camacho.zamora@sergas.es (P.C.-Z.); soraya.rumbo.feal@sergas.es (S.R.-F.); pablo.aja.macaya@sergas.es (P.A.-M.); lucia.gonzalez.pinto@sergas.es (L.G.-P.); michelle.outeda.garcia@sergas.es (M.O.-G.); romina.lucia.maceiras.senande@sergas.es (R.M.); guijarro.sanchez.p@gmail.com (P.G.-S.); maria.jose.muino.andrade@sergas.es (M.J.M.-A.); ana.fernandez.gonzalez@sergas.es (A.F.-G.); alejandro.beceiro.casas@sergas.es (A.B.); german.bou.arevalo@sergas.es (G.B.); 2 CIBER de Enfermedades Infecciosas, Instituto de Salud Carlos III, Madrid, Spain; 3 NANOBIOFAR, Centre for Research in Molecular Medicine and Chronic Diseases (CiMUS), Universidad de Santiago de Compostela, Campus Vida Avenida Barcelona s/n, 15782 Santiago de Compostela, Spain; m.martinez.guitian@gmail.com; 4 Servicio de Microbiología and Instituto de Investigación Biomédica A Coruña (INIBIC), Complexo Hospitalario Universitario A Coruña, Universidade da Coruña, As Xubias 84, 15006 A Coruña, Spain; 5 Centro Singular de Investigación en Química Biolóxica e Materiais Moleculares (CiQUS), Departamento de Química Orgánica, Universidade de Santiago de Compostela, Jenaro de la Fuente s/n, 15782 Santiago de Compostela, Spain; concepcion.gonzalez.bello@usc.es

**Keywords:** OXA-10, Enterobacterales, carbapenem resistance

## Abstract

The OXA-10 class D β-lactamase has been reported to contribute to carbapenem resistance in non-fermenting Gram-negative bacilli; however, its contribution to carbapenem resistance in Enterobacterales is unknown. In this work, minimum inhibitory concentrations (MICs), whole genome sequencing (WGS), cloning experiments, kinetic assays, molecular modelling studies, and biochemical assays for carbapenemase detection were performed to determine the impact of OXA-10 production on carbapenem resistance in two XDR clinical isolates of *Escherichia coli* with the carbapenem resistance phenotype (ertapenem resistance). WGS identified the two clinical isolates as belonging to ST57 in close genomic proximity to each other. Additionally, the presence of the *bla*_OXA-10_ gene was identified in both isolates, as well as relevant mutations in the genes coding for the OmpC and OmpF porins. Cloning of *bla*_OXA-10_ in an *E. coli* HB4 (OmpC and OmpF-deficient) demonstrated the important contribution of OXA-10 to increased carbapenem MICs when associated with porin deficiency. Kinetic analysis showed that OXA-10 has low carbapenem-hydrolysing activity, but molecular models revealed interactions of this β-lactamase with the carbapenems. OXA-10 was not detected with biochemical tests used in clinical laboratories. In conclusion, the β-lactamase OXA-10 limits the activity of carbapenems in Enterobacterales when combined with low permeability and should be monitored in the future.

## 1. Introduction

β-lactam antibiotics are therapeutic mainstays in the treatment of severe Gram-negative infections, with carbapenems often being considered first-line agents for treating infections in critically ill patients [1]. However, the therapeutic usefulness of these drugs against Gram-negative bacteria, particularly Enterobacterales, is currently at serious risk due to the increasing emergence of complex and efficient resistance mechanisms. Among the multiple mechanisms described, carbapenemase production is the most prevalent strategy by which Enterobacterales limit the effectiveness of carbapenems [2]. Carbapenemases usually display a wide spectrum of activity, are frequently co-located in promiscuous mobile genetic elements (e.g., plasmids and transposons) with other antimicrobial resistance determinants (such as ESBLs and aminoglycoside-modifying enzymes), and are increasingly detected in multidrug (MDR) or extensively-drug resistant (XDR) bacterial clones, also known as high-risk clones, which are emerging in hospitals worldwide [3,4].

KPC, OXA-48, NDM, VIM, and IMP-type enzymes are the most widespread carbapenemases in Enterobacterales and are considered by far the leading cause of carbapenem resistance [5]; however, other weaker and less prevalent enzymes may also be able to limit the activity of carbapenems. This is the case of the narrow-spectrum oxacillinases OXA-2 and OXA-10, which have received less attention than the much more ubiquitous class A, B and OXA-48 type carbapenemases, although their ability to confer carbapenem resistance when produced by the low-permeability outer membrane non-fermenters *Pseudomonas aeruginosa* and *Acinetobacter baumannii* has been extensively reported [6,7,8]. However, the specific contribution of OXA-2 and OXA-10 enzymes, if any, to the carbapenem resistance phenotype of Enterobacterales has scarcely been investigated, even though these enzymes are increasingly reported worldwide in association with high-risk clones of *Klebsiella pneumoniae* and *Escherichia coli* [9]. In this regard, Iovleva et al. recently demonstrated that carbapenem resistance in clinical *K. pneumoniae* isolates belonging to the ST258 high-risk clone and causing invasive infections was attributed to production of the OXA-2 enzyme [10]. In the present study, we performed a comprehensive characterization of two clinical *E. coli* isolates whose decreased carbapenem susceptibility is associated with OXA-10 production, and we used a molecular, biochemical, and structural approach to understand the role of this class D β-lactamase in the acquisition of carbapenem resistance in Enterobacterales.

## 2. Results and Discussion

### 2.1. Clinical Data, Resistance Phenotypes and WGS-Guided Detection of SNPs and Resistance Mechanisms

XDR *E. coli* isolate 52188484 was recovered on 24 October 2021 from a blood culture of a patient hospitalized in the Gastroenterology department of a third-level teaching hospital in Northwest Spain (University Hospital A Coruña, Northwest Galicia). As shown in Table 1, the isolate showed resistance to piperacillin, piperacillin/tazobactam, cefotaxime, ceftazidime, aztreonam, cefepime, and ertapenem, but retained susceptibility to ceftazidime/avibactam, imipenem, imipenem/relebactam, meropenem, and meropenem/vaborbactam. A second *E. coli* isolate (52190692) was recovered, nine days later (2 November 2021), from a pericholecystic abscess in another patient admitted to the same unit. This latter *E. coli* isolate showed a similar susceptibility phenotype as *E. coli* 52188484 (including carbapenem resistance), thus suggesting that the isolates were clonally related. Presumptive detection of carbapenemases using conventional hydrolytic, immunochromatographic and molecular methods for carbapenemase detection persistently yielded negative results, suggesting that ertapenem resistance was due to carbapenemase-independent mechanisms. Thus, at this point, to ascertain whether the isolates were clonally related, and to unveil the precise mechanisms of resistance involved in their susceptibility phenotypes, both isolates were subjected to whole genome sequencing (WGS).

MLST analysis using WGS data revealed that both isolates belong to the ST57 clone, which has previously been associated with transmission events linked to companion animals and animal husbandry and food-producing animals from different countries and regions [11,12]. More specifically, analysis at the SNP level revealed that both isolates were very similar, with a genomic distance of 7 SNPs. Based on the cut-off value indicated by Roer et al., which considers ≤10 SNPs the maximum likelihood cut-off for indicating short-term transmission events and outbreaks [13], these isolates can be considered almost identical at the genomic level. Thus, SNP comparison clearly indicates that transmission of XDR *E. coli* occurred through direct contact between patients or due to exposure to the same contaminated source or fomite. Furthermore, analysis of horizontally acquired determinants revealed the presence of CTX-M-65, a CTX-M-14-derived extended-spectrum β-lactamase, thus providing a good explanation for the cephalosporin resistance phenotype observed in both isolates. In accordance with previous carbapenemase detection assays, classic (e.g., *bla*_KPC_, *bla*_OXA-48_, *bla*_VIM_, *bla*_IMP_, *bla*_NDM_) or less common (e.g., *bla*_GES_, *bla*_IMI_, *bla*_SME_) carbapenemase encoding genes were not found in the genome of the isolates, suggesting carbapenem resistance was not due to a well-known carbapenemase and other mechanisms may be involved, such as the potential combination of plasmid-encoded β-lactamase with a decrease in membrane permeability [14]. However, we paid special attention to the *bla*_OXA-10_, which was also present in both isolates. OXA-10 is a classic class D β-lactamase that has previously been found to show weak carbapenemase activity and to be able to confer meropenem resistance when expressed in low permeability backgrounds, such as in *P. aeruginosa*, which is the main pathogen in which this enzyme is most commonly detected [15,16]. Finally, analysis of the chromosomally encoded β-lactam resistance mechanisms revealed the presence of inactivating mutations in the key porins OmpF (Q84stop) and OmpC (G83frameshift). These porins are involved in carbapenem uptake and known to significantly raise carbapenem MICs when associated with certain β-lactamases [17]. Thus, these findings led us to hypothesize that the clinical resistance to ertapenem, and the increased imipenem and meropenem MICs, found in the *E. coli* 52188484 and 52190692 were due to concomitant production of OXA-10 β-lactamase in combination with disruption of the OmpF and OmpC porins.

### 2.2. Role of OXA-10 on β-Lactam Resistance: Comparison with the Widespread OXA-48 Carbapenemase and Impact on Carbapenem MICs in Relation to Low and High Permeability Rates

The *bla*_OXA-10_ and *bla*_OXA-48_ genes were cloned in parallel in both *E. coli* TG1 and *E. coli* HB4 (OmpF and OmpC-deficient) reference strains to precisely determine the role of the OXA-10 enzymes on β-lactam resistance and to compare the impact of its production in the MIC of carbapenems with the widespread OXA-48 enzymes. Comparative MIC data for the respective transformants are included in Table 2. Production of OXA-10 in *E. coli* TG1 caused a substantial 256-fold increase in MICs of piperacillin and piperacillin/tazobactam, a 32-fold increase in the MIC of aztreonam, and an 8-fold increase in the MICs of cefepime, ertapenem, meropenem, and meropenem/vaborbactam. Interestingly, addition of new β-lactamase inhibitors relebactam or vaborbactam did not restore the activity of imipenem or meropenem MICs, respectively. No significant changes were identified in the other substrates.

Compared to *E. coli* TG1, production of the OXA-10 enzyme in a porin-deficient *E. coli* HB4 background significantly increased the MICs of the majority of the β-lactams (mostly due to the intrinsic higher resistance of *E. coli* HB4 strain). This effect was particularly noteworthy in the MICs of aztreonam (MIC shift from 2 to 16 mg/L), cefepime (MIC shift from 0.5 to 8 mg/L), ertapenem (MIC shift from 0.12 to 1 mg/L), and meropenem (MIC shift from 0.12 to 4 mg/L). These findings suggest that the effect of OXA-10 production in *E. coli* extends beyond classic penicillins (piperacillin) and penicillin/β-lactamase inhibitors (piperacillin/tazobactam), being able to limit the effectiveness of broad spectrum β-lactams, including carbapenems; however, the effect on carbapenem resistance seems to be influenced by low permeability.

Cloning and expression of the *bla*_OXA-48_ revealed that both enzymes have a similar effect on the MICs of piperacillin, piperacillin/tazobactam, and third- and fourth-generation cephalosporins (cefotaxime, ceftazidime, and cefepime) in both *E. coli* TG1 and HB4 backgrounds; however, major differences between the two enzymes were noted for aztreonam and carbapenems, with OXA-10 conferring higher aztreonam MICs but lower carbapenem MICs than OXA-48. Like OXA-10, the OXA-48 also showed resistance to inhibition to recently developed inhibitors relebactam and vaborbactam, since the inhibitors did not significantly improve the activity of carbapenems. Interestingly, in contrast to what has been observed for OXA-10, in which there is a proportional increase in the MIC of β-lactams in *E. coli* HB4 relative to *E. coli* TG1, OXA-48 production in the porin-deficient strain exhibited a synergistic effect which caused a substantial increase in the MICs of most carbapenem substrates. Of note, this effect led to clinical resistance to imipenem, ertapenem, meropenem, and the new combinations imipenem/relebactam and meropenem/vaborbactam, in sharp contrast to its permeable *E. coli* TG1 counterpart, which displayed susceptibility to most of these agents, except for ertapenem. Of note, our results are consistent with those of Oueslatti et al. [17], who reported that the synergy between OXA-48 production and low-permeability yielded ertapenem MICs up to 256 mg/L, in contrast to the 0.5 mg/L obtained for this antimicrobial in a host with functional OMPs (*E. coli* TOP10). Thus, these findings highlight the importance of porin deficiency in combination with class D β-lactamases which are able to interact with carbapenem substrates.

### 2.3. Hydrolytic Features of OXA-10 Enzymes against Carbapenems: Comparative Analysis with the OXA-48 Carbapenemase

OXA-10 and OXA-48 were purified and steady-state kinetics were determined to decipher and compare their relative biochemical properties (Table 3). Consistent with the MIC data, kinetic assays with imipenem and meropenem (which are first-lane agents for the combat against severe infections caused by Enterobacterales) revealed major differences between the two enzymes. OXA-10 showed detectable but very low rates of hydrolysis against both imipenem and meropenem, with *k*_cat_/*K*_m_ values of 0.0015 µM^−1^ s^−1^ and 0.0012 µM^−1^ s^−1^, respectively. These results are similar to the kinetic data obtained with purified OXA-10 β-lactamase in previous research, such as that performed by Kotsakis et al. [18], in which the evolutionary potential toward enhanced carbapenem-hydrolysing activity was assessed in comparative experiments with the native OXA-10 and different variants.

On the other hand, OXA-48 yielded higher coefficients for both agents tested (0.220 µM^−1^ s^−1^ for imipenem and 0.0019 µM^−1^ s^−1^ for meropenem), leading to a relative difference in *k*_cat_/*K*_m_ values of 147 times for imipenem and at least 1.6 times for meropenem relative to OXA-10, thus confirming that the epidemic OXA-48 displays enhanced catalytic efficiency against the most commonly used carbapenem antibiotics, especially imipenem. More specifically, these comprehensive kinetic assays help us to better understand the key differences in the activity of both carbapenems observed in the comparative MIC assays with the recombinant *E. coli* strains, (1) the negligible effect on the imipenem MIC observed with OXA-10-producing transformants (1-fold difference in imipenem MIC) is probably sustained by its poor *k*_cat_/*K*_m_ values, (2) the smaller differences between the MIC of meropenem between the recombinant OXA-10 and OXA-48 producing *E. coli* isolates (same value in TG1 and only an 8-fold difference in HB4) is probably explained by the slight differences in the affinity of the enzymes for this substrate (40.8 µM for OXA-10 versus 24.38 µM for OXA-48) and by the almost identical *k*_cat_ values (0.049 s^−1^ for OXA-10 and 0.046 s^−1^ for OXA-48). Similar results were obtained by Antunes et al. [6].

### 2.4. Structural Insights into the Interaction between the OXA-10 Enzyme and Carbapenems

**Figure 1:**
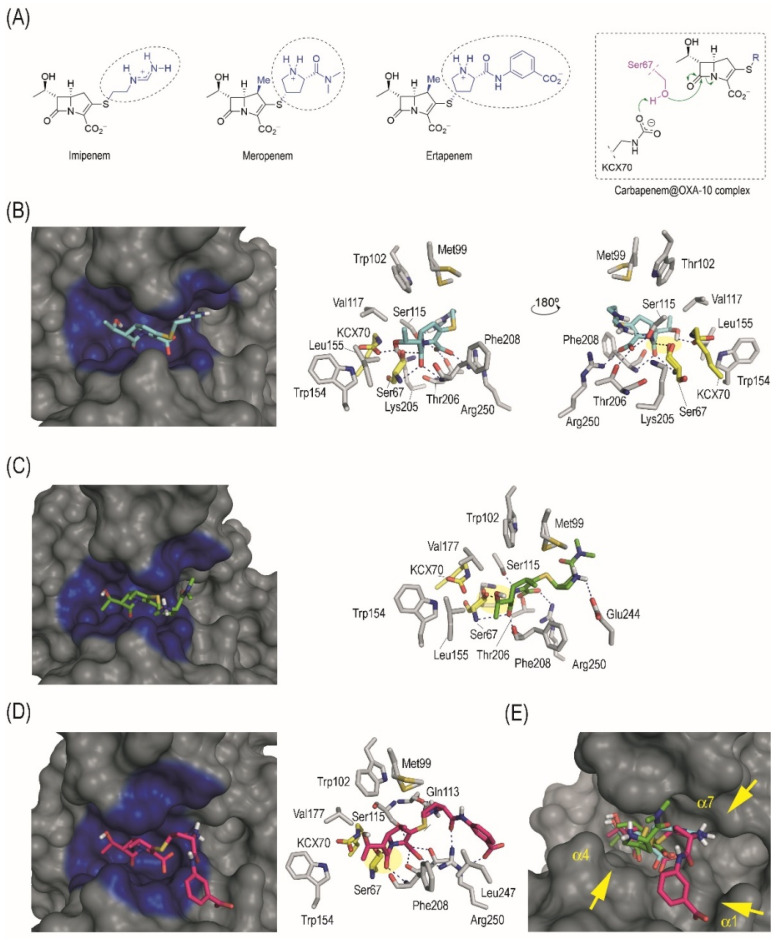
(**A**) Chemical structures of imipenem, meropenem, and ertapenem, in which the main differences are highlighted (blue with a dashed circle, R2 group), and schematic representation of the Michaelis complex that triggers the enzyme adduct formation. (**B**–**D**) Binding modes of imipenem (**B**), meropenem (**C**), and ertapenem (**D**) in the active site of OXA-10 obtained by docking studies. Overall and detailed views are provided. Relevant residues and hydrogen bonding interactions (blue dashed lines) are highlighted. Note the appropriate arrangement and close proximity of the lactam carbonyl group to the catalytic serine (Ser67) for nucleophilic attack (yellow shading). (**E**) Comparison of the overall binding mode of imipenem, meropenem, and ertapenem in the active site of OXA-10. Note how the arrangement of the carbapenem core of both antibiotics is similar, while only differences in the arrangement of the long side chain (R2 group) are observed. For imipenem, the R2 moiety interacts with the site adjacent to the catalytic centre (helix α7), while for meropenem and ertapenem, the contacts are with residues located in helix α4 and α1, respectively.

The binding mode of imipenem, meropenem, and ertapenem in the enzyme active site was explored by molecular docking to obtain some insight into the molecular causes of the hydrolysing activity of the OXA-10 enzyme against these carbapenems. From the molecular point of view, the main differences between these drugs lie in the contacts promoted by its flexible side chain (R2 group), because the position and arrangement of the positively charged ammonium group is distinct (Figure 1A). Thus, although in imipenem this positively charged group is located at the end of its linear chain, in meropenem and ertapenem this moiety is located on the central part of the branched chain (Figure 1A). It is therefore not surprising that our computational studies predicted a similar arrangement of both carbapenems for nucleophilic attack of the catalytic serine residue (Ser67). Thus, for the three Michaelis complexes (carbapenem@OXA-10 complex), the carbapenem core would be anchored to the active site through the same main polar interactions (Figure 1B–D), (i) several electrostatic and hydrogen bonding interactions between the carboxylate group and the side chain of residues Arg250, Ser115, and Thr206; (ii) the lactam carbonyl group in the carbapenem is fixed in the vicinity of the catalytic serine by two hydrogen bonding interactions with the main amide groups of Ser67 and Phe208; and (iii) the secondary hydroxyl group (R1 group) interacts by hydrogen bonding with the carbamylated form of the catalytic Lys70 residue (KCX70) [16]. Thus, the enzyme freezes the β-lactam ring of the carbapenem in a suitable arrangement for nucleophilic ring opening. Comparison of the arrangement of the three carbapenems in the active site clearly shows that the only notable differences are in the arrangement of the side chain (Figure 1E). For imipenem and meropenem, the contribution to the overall binding of the latter motif seems to be similar, as it involves one polar contact in each case. For the imipenem@OXA-10 enzyme complex, the side chain is located in close contact with the inner part of the adjacent site to the catalytic centre (Ser115) to establish a hydrogen bonding interaction, while for the meropenem@OXA-10 enzyme complex, the R2 group interacts by hydrogen bonding with the carboxylate group in Glu244. These outcomes explain the lack of important differences in the *K*_m_ values of imipenem and meropenem. Even though ertapenem only differs from meropenem in the substituent of the terminal amide group (benzoate vs. methyl groups, respectively) (Figure 1A), the disposition of the pyrrolydinyl group for both carbapenem@OXA-10 complexes are also different, which would be induced by the electrostatic interaction of the benzoate group in ertapenem with the guanidinium group of residue Arg250.

### 2.5. Detection of Carbapenemase Activity

The detection tests-based β-lactam hydrolysis were evaluated to assess their ability to detect both OXA-10 and OXA-48 when expressed in *E. coli* recombinant strains (Table 4). As expected, and consistent with previous findings, in all cases the OXA-48 producing transformants produced positive results, thus highlighting that this carbapenemase is readily detected by all home-made (e.g., CIM) or other commercially available assays [19], despite the apparently low carbapenemase activity. However, OXA-10 producing transformants persistently yielded negative results with all tests. These findings are consistent with those of a recent study by Dabos et al., who speculated that those enzymes with an imipenem *k*_cat_/*K*_m_ coefficient <0.220 µM^−1^ s^−1^ remain undetected by most of confirmatory tests aimed at detecting carbapenemase production [20]. Moreover, OXA-10-mediated carbapenem hydrolysis was not detected even by tests that do not use imipenem as substrate, such as the CIM (which uses meropenem) or the β-carba test (which uses an unknown chromogenic β-lactam that apparently has a structure that clearly differs with imipenem as substrate). Similarly, novel techniques such as the MALDI-TOF MS MBT STAR—Carba IVD Assay also did not identify carbapenemase activity in OXA-10-producing isolates.

## 3. Materials and Methods

### 3.1. Clinical Strains

Two clinical isolates of *E. coli* collected from blood (52188484) and surgical wound (52190692) samples recovered from two patients admitted to the Gastroenterology Department of the A Coruña University Hospital (Galicia, Northwest Spain) were analysed.

### 3.2. Antimicrobial Susceptibility Testing

The MICs of piperacillin, piperacillin/tazobactam, cefotaxime, ceftazidime, ceftazidime/avibactam, aztreonam, cefepime, imipenem, imipenem/relebactam, ertapenem, meropenem, and meropenem/vaborbactam were determined in triplicate experiments by reference broth microdilution method (BMD) [21]. The EUCAST version 13.0 clinical breakpoints and guidelines (http://www.eucast.org/clinical_breakpoints/ accessed on 1 February 2023) were used for reference purposes.

### 3.3. Whole Genome Sequencing

Clinical isolates were analysed by whole genome sequencing (WGS). Briefly, total genomic DNA was obtained using a Genomic DNA Buffer Set with Genomic-Tip 20/G (Qiagen, Hilden, Germany). The DNA yield was determined using the Qubit dsDNA HS Assay Kit (Thermo Fisher, Waltham, MA, USA). Indexed paired-end libraries were generated from purified genomic DNA, with a commercial library preparation kit (Nextera XT DNA Library Preparation Kit; Illumina Inc., San Diego, CA, USA) and sequenced on an Illumina MiSeq benchtop sequencer. Low-quality short reads resulting from WGS data were removed using Trimmomatic v0.39 [22], and the high-quality de novo reads from clinical isolates were finally assembled using Unicycler v 0.4.8 [23] hybrid assembler and annotated using Prokka v1.14.6 [24].

### 3.4. Characterization of Resistance Mechanisms

To examine the underlying resistance mechanisms of the isolates, sequence reads from the clinical isolates were mapped against the antibiotic susceptible *E. coli* MG1655 reference genome, and a search for amino acid substitutions or major inactivating changes was made via variant calling, performed using Snippy v4.6.0 [25]. The genes coding for the following proteins involved in β-lactam resistance were analysed in-depth, including ompC, ompF, phoE and tolC (porins), transcriptional regulators of the AcrAB-TolC efflux pump (*marR* and *tetR*/*acrR* regulators), PBP2 (*mrdA*), and PBP3 (*ftsI*). Additionally, the presence of horizontally acquired resistance determinants was predicted from de novo assemblies by accessing online databases (https://cge.cbs.dtu.dk//services/ResFinder/ accessed on 1 May 2022). Circular genome comparison of clinical strains with reference strain MG1655 were also performed using the BLAST Ring Image generator (BRIG) (Supplementary Material Figure S1) [26]. Genomic islands of *ompF* and *ompC* genes were also elucidated (Supplementary Material Figure S2).

### 3.5. Molecular Typing

STs were determined in silico by multi-locus sequence typing (MLSTs) from assembled WGS data using available online databases (https://enterobase.warwick.ac.uk/species/ecoli/ accessed on 1 May 2022). Additionally, the genetic relatedness of the isolates was analysed by matching the isolates against each other, and genetic polymorphisms (single nucleotide polymorphisms [SNPs], INDELs [insertions and deletions] or structural variants) were called using Snippy v.4.6.0 (https://github.com/tseemann/snippy accessed on 1 May 2022).

### 3.6. Cloning and Expression of bla_OXA-10_ and bla_OXA-48_ in Relation to Low and High Permeability Rates

In order to characterize the impact of OXA-10 production in β-lactam resistance in relation to low and high permeability rates, the *bla*_OXA-10_ gene from the clinical isolates and the *bla*_OXA-48_ [27] gene from a *K. pneumoniae* isolate from a previous Spanish nationwide study were expressed in *E. coli* TG1 (with functional OmpC and OmpF) and in *E. coli* HB4 (OmpC and OmpF-deficient) (note that except for the inactivation of porins OmpC and OmpF in *E. coli* HB4, both strains show baseline susceptibility to β-lactams and are not equipped with other potentially relevant β-lactam resistance mechanisms) [28]. The OXA-48 enzyme, which represents the most widespread class D carbapenemase among Enterobacterales, was cloned for comparative purposes. Briefly, *bla*_OXA-10_ and *bla*_OXA-48_ genes from the clinical isolates were amplified with primer pairs designated for this purpose, *bla*_OXA-10_-pUCP24-F-EcoRI (5′-CCGGAATTCCGGGTTAGGCCTCGCCGAAGC-3′), *bla*_OXA-10_-pUCP24-R-BamHI (5′-CGGGATCCCGTTAGCCACCAATGATGCCC-3′), *bla*_OXA-48_-F-EcoRI (5′-GGAATTCCGCATCACCAAGAATGTTGTAG-3′), and *bla*_OXA-48_-R-BamHI (5′-CGGGATCCCGCGCTAACCACTTCTAGGGAA-3′). Purified amplicons were ligated to the pUCP24 plasmid, electroporated in parallel into *E. coli* TG1 and *E. coli* HB4, and plated on 10 mg/L gentamicin-LB agar plates. The MICs of β-lactams were determined for the recombinant isolates following the above-described methodology Section 3.2.

### 3.7. Protein Purification

*bla*_OXA-10_ and *bla*_OXA-48_ genes from the clinical strains were cloned into the p-GEX-6P-1 plasmid using primers *bla*_OXA-10_-pGEX-F-BamHI (5′-CGCGGATCCGCGGGTTCAATTACAGAAAATACG-3′) and *bla*_OXA-10_-pGEX-R-EcoRI (5′-CGCGAATTCCGGTTAGCCACCAATGATGCCC-3′) for OXA-10, and *bla*_OXA-48_-pGEX-F-BamHI (5′-AAA GGATCCAAGGAATGGCAAGAAAACAAA-3′) and *bla*_OXA-48_-pGEX-R-EcoRI (5′-AAAGAATTCCTAGGGAATAATTTTTTCCTGTTT-3′). The resulting amplicons were digested with BamHI and EcoRI, ligated to p-GEX-6P-1 and electroporated into the protease-deficient *E. coli* BL21 to generate the fusion proteins glutathione S-transferase (GST)—OXA-10 and glutathione S-transferase (GST)—OXA-48. These recombinant proteins were purified to homogeneity using the GST gene fusion system (Amersham Pharmacia Biotech, Europe) according to the manufacturer’s instructions. Then, the GST tag was cleaved off, and the resulting OXA-10 and OXA-48 proteins were obtained. Finally, SDS-PAGE was performed to ascertain the absence of impurities in the final extract (99% purity).

### 3.8. Steady-State Kinetics

The kinetic parameters of purified OXA-10 and OXA-48 β-lactamases were determined at room temperature in a Nicolet Evolution 300 spectrophotometer (Thermo Fisher Scientific, Waltham, MA, USA) and an Epoch 2 Microplate Spectrophotometer (Biotek, Winooski, VT, USA). Each experiment was performed in triplicate with 50 mM sodium phosphate and 20 mM sodium bicarbonate in 0.2-cm-pathlength cuvettes. Kinetic parameters were determined by measuring the initial velocity rates. For this purpose, the kinetic data were fitted to the Michaelis–Menten equation and *V_max_, k*_cat_ and *K*_m_ values were calculated, as previously described [29]. The wavelengths (λ) and absorption coefficients (ε) used for each antibiotic were as follows: λ = 297 nm and ε = 9210 M^−1^ cm^−1^ for imipenem, λ = 297 nm and ε = 10,940 M^−1^ cm^−1^ for meropenem.

### 3.9. Molecular Modelling Studies

The binding mode of the imipenem, meropenem, and ertapenem in the OXA-10 enzyme active site was explored by molecular docking using the GOLD program [30], version 2020.2, following our previously described protocol [31]. The protein coordinates were taken from the crystal structure of the OXA-10 enzyme covalently modified by a cyclobutanone β-lactam mimic (PDB 3LCE, 2.0 Å) [32]. Figures depicting structures were prepared using PYMOL software, version 1.5. ChemDraw software version 22.0.0.22 was used to draw chemical structures (http://www.perkinelmer.co.uk/category/chemdraw accessed on 15 September 2022).

### 3.10. Biochemical Detection of Carbapenemase Activity

Carbapenemase activity was detected in clinical and recombinant isolates using 5 different methodologies, (1) the Carba NP test [33], (2) the CIM test [34], (3) the modified Hodge test [35], (4) the β-carba test (Bio-Rad, Hercules, CA, USA) [36], and the (5) MALDI-TOF MS MBT STAR—Carba IVD Assay [37] in accordance with the manufacturer’s recommendations.

### 3.11. Nucleotide Accession Numbers

The WGS data of the *E. coli* strains 52188484 and 52190692 have been deposited in Genbank databases under accession number PRJNA934346 (bioproject). The WGS data of reference strains *E. coli* TG1, *E. coli* HB4 and *E. coli* MG1655 is also available at Genbank databases under accession numbers: PRJNA514245 (*E. coli* TG1), PRJNA688628 (*E. coli* HB4), and SAMN02604091 (*E. coli* MG1655).

## 4. Conclusions

The oxacillinase enzyme OXA-10 is increasingly found in high-risk clones of the Enterobacterales species, such as *K. pneumoniae* and *E. coli*, but its impact on the β-lactam resistance profile of these growing clinical threats has remained unexplored to date. In this study, we provide a detailed analysis of two clonally related *E. coli* clinical isolates involved in life-threatening infections and bearing this enzyme. Using WGS, cloning experiments, biochemical studies, molecular modelling, and hydrolysis tests, we dissected the specific contribution of this challenging resistance determinant on a growing clinical challenge, carbapenem resistance. Based on WGS data obtained from the clinical isolates, and the findings obtained with recombinant strains, we hypothesise that the production of the OXA-10 enzyme in combination with the inactivation of the outer membrane porins OmpF and OmpC can increase carbapenems MICs. In addition to this, the low carbapenem hydrolysing activity obtained in kinetic assays and persistent negative results in carbapenemase detection methods also indicate that OXA-10 enzyme should not be considered a carbapenemase, even though modelling assays highlight that the enzyme recognizes carbapenem substrates. Furthermore, these results support the high activity of imipenem and meropenem against *E. coli* isolates, as even with OXA-10 production and low membrane permeability the MIC values remain below the resistance breakpoints for both antibiotics. Altogether, our findings help to expand our understanding of the OXA-10 enzyme on β-lactam resistance and specifically in carbapenem resistance. The potential of this resistance determinant to spread among clinically relevant Enterobacterales clones and species warrants maintaining surveillance of this resistance determinant in the future.

## Figures and Tables

**Table 1:** Demographic, phenotypic, and genotypic features of *E. coli* clinical isolates 52188484 and 52190692.

				MIC (mg/L)												
Strain	Isolation Date	Source	ST	PIP (R > 8)	P/T (R > 8)	CTX (R > 2)	CAZ (R > 4)	CZ/A (R > 8)	AZT (R > 4)	FEP (R > 4)	IPM (R > 4)	IM/R (R > 2)	ERT (R > 0.5)	MRP (R > 8)	M/V (R > 8)	β-Lactam Resistance Genotype
*E. coli* 52188484	24 October 2021	Blood	ST57	≥512	128	512	16	0.25	512	64	0.5	0.25	2	2	1	*bla*_OXA-10_, *bla*_CTX-M-65_, *ompC* G83frameshift, *ompF* Q84stop codon
*E. coli* 52190692	2 November 2021	Surgical wound	ST57	≥512	128	256	16	0.25	512	128	0.5	0.25	2	4	1	*bla*_OXA-10_, *bla*_CTX-M-65_, *ompC* G83frameshift, *ompF* Q84stop codon

PIP, piperacillin; P/T, piperacillin/tazobactam; CTX, cefotaxime; CAZ, ceftazidime; CZ/A, ceftazidime/avibactam; AZT, aztreonam; FEP, cefepime; IPM, imipenem; IM/R, imipenem/relebactam; ERT, ertapenem; MRP, meropenem; M/V, meropenem/vaborbactam.

**Table 2:** Susceptibility profiles of the recombinant *E. coli* TG1 and *E. coli* HB4 (OmpC and OmpF-deficient) expressing *bla*_OXA-10_ and *bla*_OXA-48_ β-lactamases.

	MIC (mg/L)											
Strain	PIP (R > 8)	P/T (R > 8)	CTX (R > 2)	CAZ (R > 4)	CZ/A (R > 8)	AZT (R > 4)	FEP (R > 4)	IPM (R > 4)	IM/R (R > 2)	ERT (R > 0.5)	MRP (R > 8)	M/V (R > 8)
*E. coli* TG1	0.5	0.5	≤0.06	≤0.06	0.03	≤0.06	≤0.06	0.06	0.06	≤0.015	≤0.015	≤0.015
*E. coli* TG1 + pUCP24-*bla*_OXA-10_	128	128	0.12	0.12	0.06	2	0.5	0.06	0.06	0.12	0.12	0.12
*E. coli* TG1 + pUCP24-*bla*_OXA-48_	256	128	0.25	0.12	0.12	0.06	0.25	0.5	0.25	1	0.12	0.25
*E. coli* HB4	8	4	0.5	1	0.25	0.5	0.5	0.12	0.12	0.12	0.12	0.12
*E. coli* HB4 + pUCP24-*bla*_OXA-10_	512	512	2	1	1	16	8	0.25	0.25	1	4	4
*E. coli* HB4 + pUCP24-*bla*_OXA-48_	≥512	512	4	0.5	0.5	0.5	4	16	4	32	32	32

PIP, piperacillin; P/T, piperacillin/tazobactam; CTX, cefotaxime; CAZ, ceftazidime; CZ/A, ceftazidime/avibactam; AZT, aztreonam; FEP, cefepime; IPM, imipenem; IM/R, imipenem/relebactam; ERT, ertapenem; MRP, meropenem; M/V, meropenem/vaborbactam.

**Table 3:** Kinetic parameters of β-lactamases OXA-10 and OXA-48 for representative carbapenems.

	OXA-10			OXA-48			
Drug	*K*_m_ (µM)	*k*_cat_ (s^−1^)	*k*_cat_/*K*_m_ (µM^−1^ s^−1^)	*K*_m_ (µM)	*k*_cat_ (s^−1^)	*k*_cat_/*K*_m_ (µM^−1^ s^−1^)	Ratio *k*_cat_/*K*_m_ for OXA-48/OXA-10
Imipenem	36.1 ± 14.6	0.054 ± 0.003	0.0015 ± 0.0004	7.24 ± 0.54	1.590 ± 0.119	0.220 ± 0.012	146.6
Meropenem	40.8 ± 10.9	0.049 ± 0.009	0.0012 ± 0.0002	24.38 ± 1.80	0.046 ± 0.004	0.0019 ± 0.0001	1.582

Data represent the means from three independent experiments.

**Table 4:** Diagnostic biochemical tests aimed at detecting carbapenemase activity performed on clinical and recombinant *E. coli* isolates expressing *bla*_OXA-10_ and *bla*_OXA-48_ enzymes.

Isolate	Carba NP Test	CIM Test	Modified Hodge Test	Β-Carba Test	MALDI-TOF MS MBT STAR—Carba IVD Assay
*E. coli* 52188484	−	−	−	−	−
*E. coli* 52190692	−	−	−	−	−
*E. coli* TG1 + pUCP24-*bla*_OXA-10_	−	−	−	−	−
*E. coli* HB4 + pUCP24-*bla*_OXA-10_	−	−	−	−	−
*E. coli* TG1 + pUCP24-*bla*_OXA-48_	+	+	+	+	+
*E. coli* HB4 + pUCP24-*bla*_OXA-48_	+	+	+	+	+

Key: +, positive test result; −, negative test result.

## Data Availability

The data presented in this study are available on request from the corresponding author. The data are not publicly available due to privacy.

## Supplementary Materials

The following supporting information can be downloaded at: https://www.mdpi.com/article/10.3390/antibiotics12060999/s1, Figure S1: Circular genome comparison of clinical *E. coli* strains 52188484 and 52190692 with reference strain MG1655; Figure S2: Genomic island of *ompC* and *ompF* genes in the two clinical *E. coli* isolates 52188484 and 52190692.
