# Supplementary material for: Interplay between OXA-10 β-Lactamase Production and Low Outer-Membrane Permeability in Carbapenem Resistance in Enterobacterales
Source: Antibiotics (Basel). 2023 Jun 1;12(6):999. doi: 10.3390/antibiotics12060999 (PMC10295649; doi:10.3390/antibiotics12060999)

**Supplementary Material.**

**Figure S1. Circular genome comparison of clinical *E. coli* strains 52188484 and 52190692 with reference strain MG1655.**

The external line represents the reference genome of strain *E. coli* MG1655. Grey blocks indicate the genes of the reference strain. Blue and red lines indicate the genomes of strains 52188484 and 52190692 respectively. Orange/grey peaks indicate GC skew. Green/blue peaks indicate the GC variation according to the average GC content.

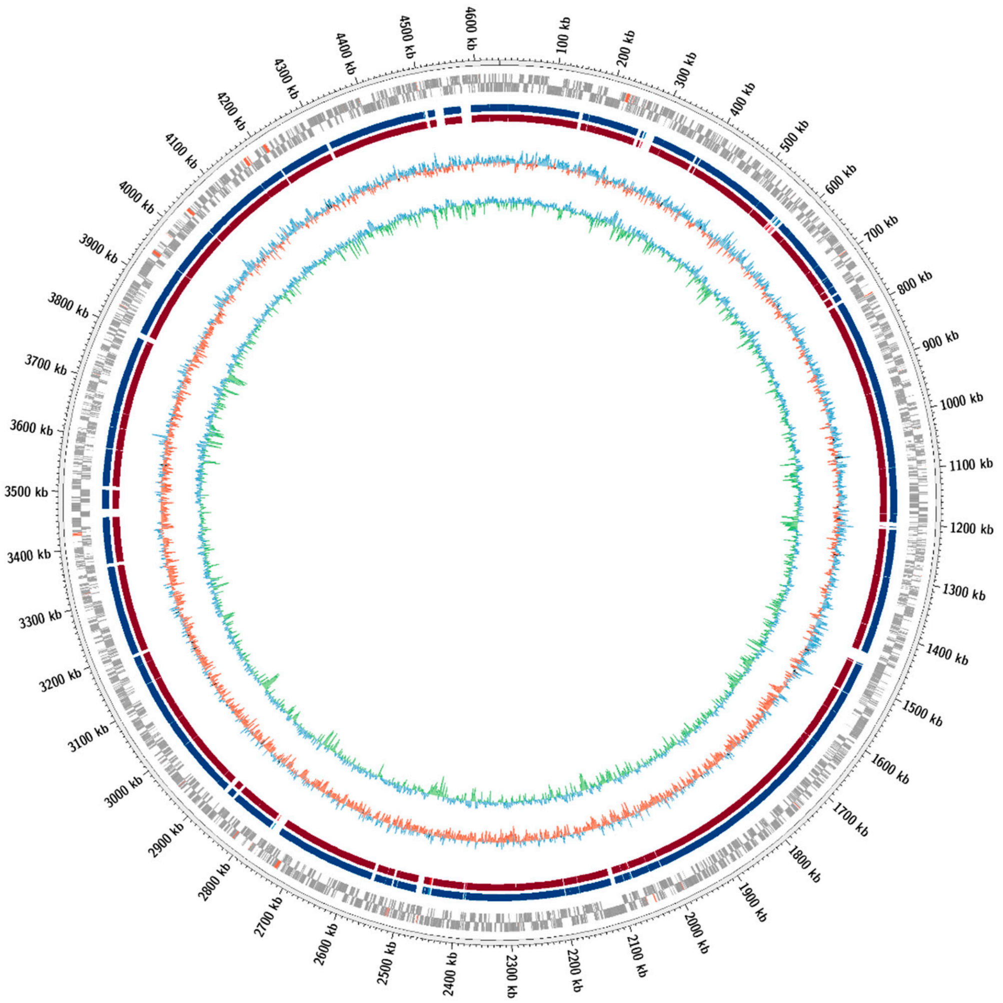

**Figure S2**. **Genomic island of *ompC* and *ompF* genes in the two clinical *E. coli* isolates 52188484 and 52190692.**

A) Genomic island of *ompC* gene. Yellow indicates the hypothetical *ompC* area, the disruption in the two clinical strains is shown below.

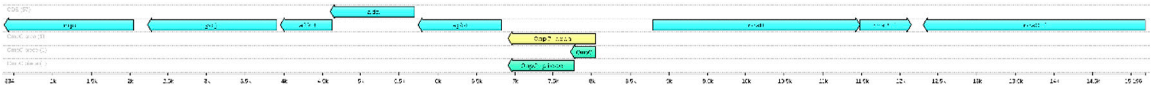

B) Genomic island of *ompF* gene. Yellow indicates the hypothetical *ompF* area, the disruption in the two clinical strains is shown below.

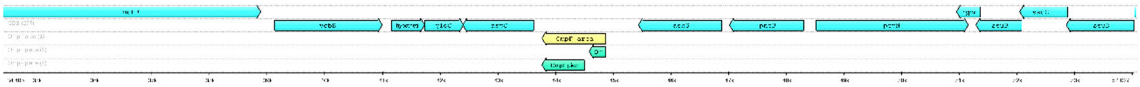

Supplement: Supplementary file 1 [file antibiotics-12-00999-s001.zip › antibiotics-2304861-supplementary.pdf]
